# The Energy Compensation of the HRG Based on the Double-Frequency Parametric Excitation of the Discrete Electrode

**DOI:** 10.3390/s20123549

**Published:** 2020-06-23

**Authors:** Wanliang Zhao, Hao Yang, Fucheng Liu, Yan Su, Lijun Song

**Affiliations:** 1 School of Mechanical Engineering, Nanjing University of Science and Technology, Nanjing 210094, China; zhaodada999@163.com; 2 Shanghai Aerospace Control Technology Institute, Shanghai 201100, China; 18810450298@139.com (H.Y.); liufch@126.com (F.L.); 3 School of Information & Control Engineering, Xi’an University of Architecture and Technology, Xi’an 710055, China; songlijun9071@sina.com

**Keywords:** hemispherical resonator gyroscopes (HRG), whole-angle control, parametric excitation, double-frequency parametric excitation, discrete electrode

## Abstract

In this study, for energy compensation in the whole-angle control of Hemispherical Resonator Gyro (HRG), the dynamical equation of the resonator, which is excited by parametric excitation of the discrete electrode, is established, the stability conditions are analyzed, and the method of the double-frequency parametric excitation by the discrete electrode is derived. To obtain the optimal parametric excitation of the resonator, the total energy stability of the resonator is simulated for the evolution of the resonator vibration with different excitation parameters and the free precession of the standing wave by the parametric excitation. In addition, the whole-angle control of the HRG is designed, and the energy compensation of parametric excitation is proven by the experiments. The results of the experiments show that the energy compensation of the HRG in the whole-angle control can be realized using discrete electrodes with double-frequency parametric excitation, which significantly improves the dynamic performance of the whole-angle control compared to the force-to-rebalance.

## 1. Introduction

The hemispherical resonator gyro (HRG) is a kind of vibratory gyroscope; it measures the gyro rotation by the precession effect of a vibration standing wave and has the advantages of high accuracy, high reliability, and long life. With an increasing number of applications of the HRG in aerospace, navigation, tactics and other fields, the HRG has become a focus in the field of inertial navigation technology [1,2,3,4].

Control mode options of the HRG are mainly the force-to-rebalance and the whole-angle control. The HRG is a rate gyro in the force-to-rebalance mode [1,2]. When the carrier platform rotates, an electrostatic control force is used to keep the vibration standing wave azimuth and carrier platform relatively stationary, which is proportional to the carrier angular velocity. By measuring the control force, the carrier angular velocity will be obtained. The HRG is an integrated gyro in the whole-angle control, and the angle signal is directly output. In theory, the dynamic range is infinite when it is working under the whole-angle control, and linearity is extremely high, that is, better than 1 ppm [5,6]. This working model has hold function when the power is disconnected, and the HRG maintains an effective working time of 10–20 min after power-off. This is an important attribute of the HRG, allowing it to achieve the performance of the inertial stage.

The dynamic range of the HRG with traditional force-to-rebalance is only about ±10°/s [7], and its scale factor is easily affected by temperature [8]. Thus, it cannot meet application requirements in many fields. Therefore, the whole-angle control with infinite dynamics has become the main technical approach to achieve high precision, high stability, and high reliability.

Due to damping and frequency splitting, there are two main control loops in the whole-angle control: the energy compensation loop and the quadrature control loop. The energy compensation loop is used to overcome the effect of damping and maintain the amplitude of the resonator to a constant level. Then, the quadrature control loop is used to suppress the traveling wave components in the vibration. With the energy compensation loop and the quadrature control loop, a pure standing wave with constant amplitude will be established in the resonator and the HRG works as a rate-integrating gyro. Therefore, the energy compensation is the most fundamental technology to realize the whole-angle control. The method of energy compensation in whole-angle control is more complex and more difficult than that of force-to-rebalance because the standing wave needs to move freely in the resonator.

For the energy compensation method of the whole-angle control HRG, this study researched the energy compensation of the HRG based on the double-frequency parametric excitation of the discrete electrode. In this paper, the dynamic equation of the HRG excited by the double-frequency parametric excitation of the discrete electrode is established, and the stable boundary conditions of the parametric excitation are analyzed. The optimal excitation method of the resonator is determined by simulation of the evolution with different excitation parameters, and a practical system is built to verify the energy compensation of the resonator by the double-frequency parametric excitation of the discrete electrode.

## 2. The Theory of Parametric Excitation of the Discrete Electrode

### 2.1. The Parametric Excitation Method

The loss of energy of the HRG is mainly due to the internal loss of the resonator material and the influence of residual gas in the instrument. Many excitation methods can be used to compensate this loss of energy. Zhao H B. et al. used position excitation to study the energy compensation of the force-to-rebalance [9], and found that the position excitation system is not suitable for the amplitude profile of whole-angle control, because the standing wave azimuth will be bound to the excitation electrode. Gregory, J.A. et al. described a vector synthesis of force based on the position excitation to realize the energy compensation of whole-angle control [10]. This method can effectively compensate for the energy loss of the hemispherical resonator. However, because the phase of excitation electrode and the readout electrode is not ideal, a large error will be caused by the mutual interference of the control circuit, thus, affecting the accuracy of the device. Parametric excitation was first proposed to control the energy of the HRG in references [11,12], which was supported by the ring electrode surrounding the cover of the three-suite configuration. It is a double frequency scheme (compared with the vibration frequency of the resonator). However, the study lacked theoretical analysis. Based on previous studies, Matveyev V et al. proposed a parametric excitation of the single frequency, and this method was described in detail [13], a ring electrode is used to apply the excitation signal, and the frequency of the excitation signal is the same as the frequency of the resonator, however, there was large loop crosstalk caused by the excitation signal. At the same time, if there is an uneven gap in the structure of the resonator and the ring electrode, it will introduce a larger position excitation (the excitation system is a combination of parameter and position), and drift of the HRG will be caused by the single frequency parametric excitation.

In summary, compared with the vector synthesis of force, the parametric excitation method has the advantage of noninterference with the other control circuits. With the development of miniaturization and low cost, the configuration without the ring electrode has gradually become the mainstream. Based on the single frequency of three-suite configuration, double-frequency parametric excitation has been redesigned to achieve the advantage of the discrete electrode configuration, which has the advantages of less crosstalk in the signal detection loop and no compound excitation.

### 2.2. The Dynamic Equations of Resonators Excited by the Discrete Electrode

The whole-angle control of the HRG is based on the principle that the sensitive electrode converts the physical vibration signal into an electrical signal. Information relating to vibration amplitude, vibration frequency and precession angle is separated and solved by the signal processing algorithm, and the control algorithm is used to stabilize the resonator and ensure stable convergence of the precession angle.

**Figure 1:**
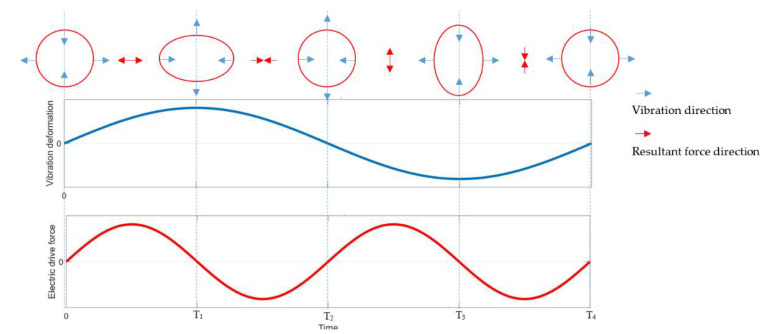
The schematic diagram of double-frequency parametric excitation.

Parametric excitation is achieved by the electrode surrounding the edge of the resonator, and the surfaces of the resonator and the electrode can be used as the capacitor plate. In order to achieve energy compensation, the direction of the electric driving force needs to be maintained in the same direction as the speed of vibration. The basic principle of double-frequency parameter excitation is explained in Figure 1. During the working process of the HRG, when the resonator is not deformed, the electric drive forces acting on the resonator are balanced; when the resonator is deformed, the resonator moves towards the direction of maximum deformation, and the resultant force of the electric driving force is always consistent with the vibration direction, so the resultant force of the electric driving force will always do positive work on the harmonic oscillator. Thus, the parametric excitation of the resonator will occur when the frequency variation is equal to twice the natural frequency of the principal mode.

**Figure 2:**
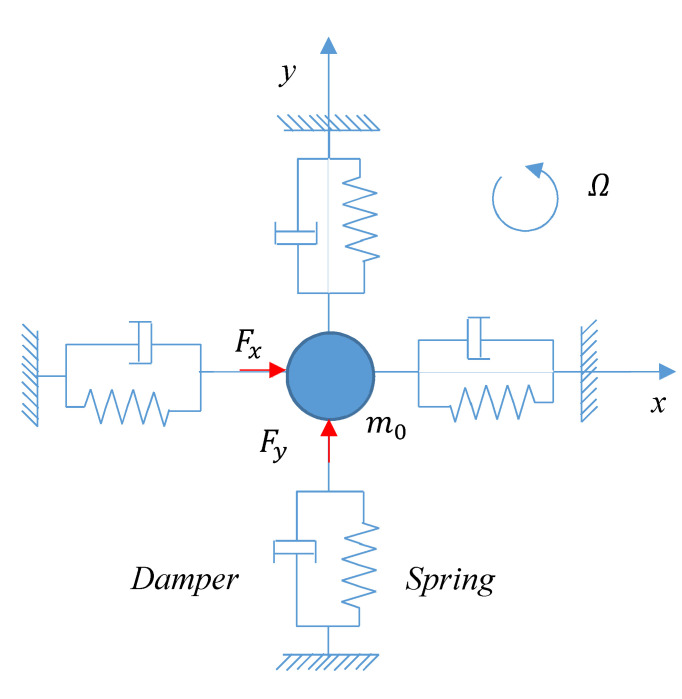
The equivalent physical model of hemispherical resonator.

If the physical model of the vibration is simplified as a spring-mass damping system, as shown in Figure 2, the dynamic model of the resonator is:

(1) $$\{\begin{array}{c} m_{0}\ddot{x}-4m_{0}K\Omega \dot{y}+\frac{m_{0}\omega_{0}}{Q_{0}}\dot{x}+m_{0}\omega_{0}^{2}x=F_{x}\\ m_{0}\ddot{y}+4m_{0}K\Omega \dot{x}+\frac{m_{0}\omega_{0}}{Q_{0}}\dot{y}+m_{0}\omega_{0}^{2}y=F_{y} \end{array}$$

where *x* and *y* are the vibration displacements on the 0° and 45° detection electrodes, and *m*_0_ is the equivalent mass of the resonator. *Ω* is the rotational speed of the platform and *K* is the precession factor; K=0.277. $\omega_{0}$ is the natural frequency of the resonator. The value $Q_{0}$ is the quality factor. $F_{x},F_{y}$ represent the equivalent force applied by the excitation electrode.

**Figure 3:**
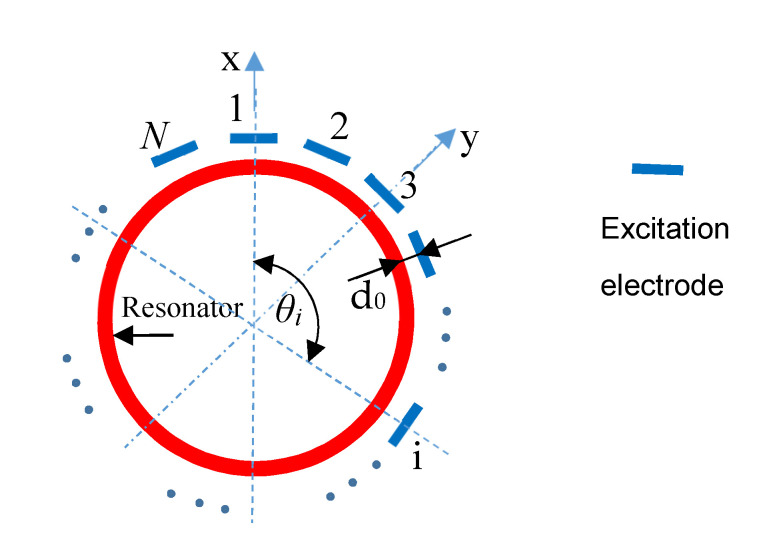
The schematic diagram of discrete excitation electrodes.

As shown in the derivation in Appendix A, from references [14,15], when the parametric excitation of the HRG has occurred, the storage charge of capacitance will change due to the displacement between the electrode plates. To control the fundamental vibrational of the resonator, the amount of charge is input into Equation (1). Thus, obtaining the force expression which depends on the voltage applied to the electrode:

(2) $$\{\begin{array}{c} F_{x}=\frac{C_{0}}{2d_{0}^{3}}\overset{N}{\underset{i=1}{\sum }}V_{i}^{2}\left(d_{0}^{2}+2d_{0}xcos2\theta_{i}+2d_{0}ysin2\theta_{i}\right)cos2\theta_{i}\\ F_{y}=\frac{C_{0}}{2d_{0}^{3}}\overset{N}{\underset{i=1}{\sum }}V_{i}^{2}\left(d_{0}^{2}+2d_{0}xcos2\theta_{i}+2d_{0}ysin2\theta_{i}\right)sin2\theta_{i} \end{array}$$

where $C_{0}$ is the capacitance which is composed of the single driving electrode and the resonator; the distribution of discrete electrodes is shown in Figure 3; $d_{0}$ is the average gap value of the single driving electrode and the resonator; *N* is the number of the excitation electrode; $\theta_{i}$ is the orientation of the *i*-th electrode, and $\theta_{i}=\frac{2\pi \left(i-1\right)}{N}$; $V_{i}^{\;}$ is the voltage of the *i*-th electrode.

When Equation (2) is substituted into Equation (1), the dynamic equation of the resonator is changed into:

(3) $$\{\begin{array}{c} \ddot{x}-4K\Omega \dot{y}+\frac{\omega_{0}}{Q_{0}}\dot{x}+\omega_{0}^{2}x=\frac{C_{0}}{2m_{0}d_{0}^{3}}\overset{N}{\underset{i=1}{\sum }}V_{i}^{2}\left(d_{0}^{2}+2d_{0}xcos2\theta_{i}+2d_{0}ysin2\theta_{i}\right)cos2\theta_{i}\\ \ddot{y}+4K\Omega \dot{x}+\frac{\omega_{0}}{Q_{0}}\dot{y}+\omega_{0}^{2}y=\frac{C_{0}}{2m_{0}d_{0}^{3}}\overset{N}{\underset{i=1}{\sum }}V_{i}^{2}\left(d_{0}^{2}+2d_{0}xcos2\theta_{i}+2d_{0}ysin2\theta_{i}\right)sin2\theta_{i} \end{array}$$

**Figure 4:**
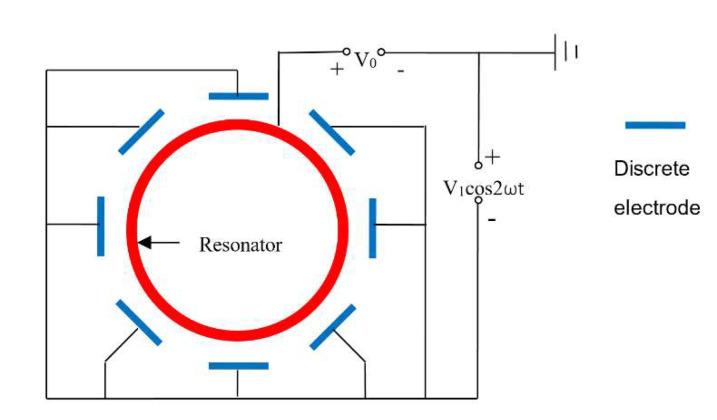
Discrete electrode distribution and electrical connection with the parametric excitation. Eight discrete electrodes are equally spaced around the resonator, and the excitation voltage frequency is twice the frequency of the resonator.

In the operation of the HRG, as shown in Figure 4, the DC bias is applied to the resonator, and the excitation voltage $V_{\;}=V_{1}\cos2\omega t$ is applied to the electrode. At this time,

(4) $$V_{i}^{2}={\left(V_{0}+V\right)}^{2}=V_{0}^{2}+2V_{0}V_{1}\cos2\omega t+{\left(V_{1}\cos2\omega t\right)}^{2}$$

As $d_{0}^{2}$ in Equation (3) and $V_{0}^{2},{\left(V_{1}\cos2\omega t\right)}^{2}$ in Equation (4) do not affect the energy compensation of the double-frequency parametric excitation (see Appendix B), the dynamic equation of the resonator with the parametric excitation is:

(5) $$\{\begin{array}{c} \ddot{x}-4K\Omega \dot{y}+\frac{\omega_{0}}{Q_{0}}\dot{x}+\omega_{0}^{2}x=\frac{C_{0}}{m_{0}d_{0}^{2}}\overset{N}{\underset{i=1}{\sum }}V_{0}V_{1}\cos2\omega t\left(2xcos2\theta_{i}+2ysin2\theta_{i}\right)cos2\theta_{i}\\ \ddot{y}+4K\Omega \dot{x}+\frac{\omega_{0}}{Q_{0}}\xi \dot{y}+\omega_{0}^{2}y=\frac{C_{0}}{m_{0}d_{0}^{2}}\overset{N}{\underset{i=1}{\sum }}V_{0}V_{1}\cos2\omega t\left(2xcos2\theta_{i}+2ysin2\theta_{i}\right)sin2\theta_{i} \end{array}$$

For *N* = 8, in which eight electrodes are excited at the same time, the vibration equation of the resonator is

(6) $$\{\begin{array}{c} \ddot{x}-4K\Omega \dot{y}+\frac{\omega_{0}}{Q_{0}}\dot{x}+\omega_{0}^{2}x=\delta \cos2\omega tx\\ \ddot{y}+4K\Omega \dot{x}+\frac{\omega_{0}}{Q_{0}}\xi \dot{y}+\omega_{0}^{2}y=\delta \cos2\omega ty \end{array}$$

where $\delta =\frac{8C_{0}V_{0}V_{1}}{m_{0}d_{0}^{2}}$.

From Equation (6), the x-mode and y-mode change periodically by the law of δcos2ωt under the driving force of double frequency on the discrete electrode. In this way, the excitation force is not applied to the resonator in the form of external force, but is indirectly realized by the periodic change of the parameters (stiffness) of the resonator. The equivalent stiffness becomes $k_{eff}=m_{0}\omega_{0}^{2}-m_{0}\delta \cos2\omega t$ and the parametric vibration of the resonator occurs [16]. The energy of the x-mode and y-mode can be compensated adaptively according to the respective amplitude, and the parametric excitation of the HRG can be realize by the discrete electrode. Moreover, the excitation frequency is twice the vibration frequency of the resonator, can effectively separate the excitation signal and the vibration signal in the frequency domain, and reduce the detection error caused by the coupling of the excitation signal.

### 2.3. The Stability Boundary of the Double-Frequency Parametric Excitation

The vibration of parametric excitation is a form of non-linear vibration. When the excitation conditions satisfied the stability boundary, the resonator can be excited by the parameters [13,16].

The solution of Equation (6) can be written as:

(7) $$\begin{array}{c} x=\mathrm{Acos}\omega t+\mathrm{Psin}\omega t\\ y=\mathrm{Bcos}\omega t+\mathrm{Qsin}\omega t \end{array}$$

During a vibration period, A,B,P,Q can be considered as constants. Then, the slow variables A(t),B(t),P(t),Q(t) can be introduced:

(8) $$\begin{array}{c} x=A\left(t\right)\cos\omega t+P\left(t\right)\sin\omega t\\ \begin{array}{c} y=B\left(t\right)\cos\omega t+Q\left(t\right)\sin\omega t\\ \begin{array}{c} \dot{x}=-A\left(t\right)\omega \sin\omega t+P\left(t\right)\omega \cos\omega t\\ \dot{y}=-B\left(t\right)\omega \sin\omega t+Q\left(t\right)\omega \cos\omega t \end{array} \end{array} \end{array}$$

If Equation (8) of the slow variable is substituted into the vibration Equation (6) and the results are averaged by the fast variable, ωt, the evolution equation of the slow variable can be obtained as follows (see Appendix C):

(9) $$\{\begin{array}{c} \dot{P}=-\frac{1}{2}\left(\Delta -\frac{1}{2}s\right)A-\frac{1}{2}\frac{\omega_{0}}{Q_{0}}P+2K\Omega Q\\ \dot{A}=-\frac{1}{2}\frac{\omega_{0}}{Q_{0}}A+\frac{1}{2}\left(\Delta +\frac{1}{2}s\right)P+2K\Omega B\\ \dot{Q}=-\frac{1}{2}\left(\Delta -\frac{1}{2}s\right)B-\frac{1}{2}\frac{\omega_{0}}{Q_{0}}Q-2K\Omega P\\ \dot{B}=-\frac{1}{2}\frac{\omega_{0}}{Q_{0}}B+\frac{1}{2}\left(\Delta +\frac{1}{2}s\right)Q-2K\Omega A \end{array}$$

where Ω = Ω(t) is a time function of slow change, and its rate of change can be ignored $;\Delta =\frac{\left(\omega_{\;}^{2}-\omega_{0}^{2}\right)}{\omega },s=\frac{\delta }{\omega }$, and δ are proportional to the amplitude of the excitation voltage,${\;\omega }_{0}^{\;}$ is the vibration frequency of the resonator, ω is half of the excitation frequency, and $\omega_{0}^{\;}\approx \omega$. The system of equations can be written as a matrix:

(10) $$\left[\begin{array}{c} \dot{P}\\ \dot{A}\\ \dot{Q}\\ \dot{B} \end{array}\right]=\left[\begin{array}{cccc} -\frac{\omega_{0}}{{2Q}_{0}} & -\frac{1}{2}(\Delta -\frac{1}{2}s) & 2K\Omega & 0\\ \frac{1}{2}(\Delta +\frac{1}{2}s) & -\frac{\omega_{0}}{{2Q}_{0}} & 0 & 2K\Omega \\ -2K\Omega & 0 & -\frac{\omega_{0}}{{2Q}_{0}} & -\frac{1}{2}(\Delta -\frac{1}{2}s)\\ 0 & -2K\Omega & \frac{1}{2}(\Delta +\frac{1}{2}s) & -\frac{\omega_{0}}{{2Q}_{0}} \end{array}\right]\left[\begin{array}{c} P\\ A\\ Q\\ B \end{array}\right]$$

When the time function of slow change is Ω = 0, the stability boundary of the equation can be obtained. For the resonator to have stable vibration, it is required that:

(11) $$\det\left[\begin{array}{cc} -\frac{1}{2}\left(\Delta -\frac{1}{2}s\right) & -\frac{1}{2}\frac{\omega_{0}}{Q_{0}}\\ -\frac{1}{2}\frac{\omega_{0}}{Q_{0}} & \frac{1}{2}\left(\Delta +\frac{1}{2}s\right) \end{array}\right]=0$$

which is

(12) $$\left(\Delta -\frac{1}{2}s\right)\left(\Delta +\frac{1}{2}s\right)+\frac{\omega_{0}^{2}}{Q_{0}^{2}}=0$$

**Figure 5:**
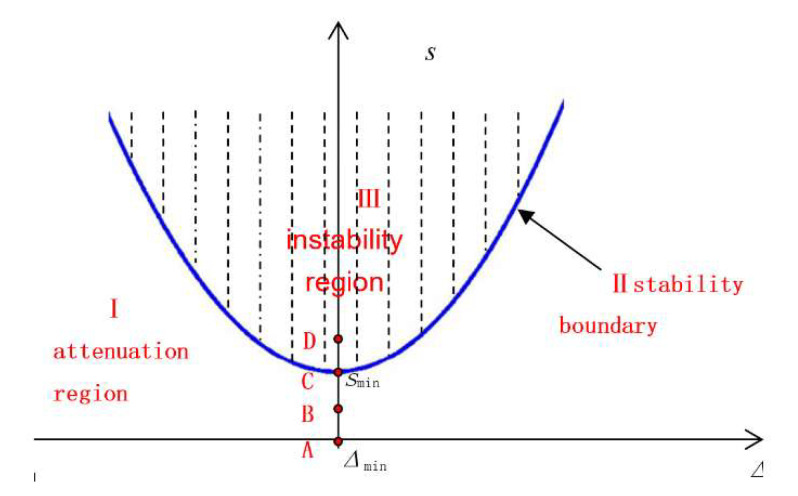
The stability boundary diagram of the parametric excitation.

Equation (12) determines the stable boundary conditions of the resonator with parametric excitation, as shown in Figure 5.

According to the different excitation parameters (Δ, s) of the HRG, the vibration state of the resonator is divided into three regions, i.e., I is the attenuation region, II is the stability boundary, and III is the instability region. When the excitation parameter is in area I, the vibration of the resonator will decrease from the initial vibration to 0; when the excitation parameter is in area III, the vibration of the resonator will increase exponentially from the initial vibration; when the excitation parameter is on boundary condition II, the resonator will maintain its initial vibration [13].

To realize the energy control of the HRG by parametric excitation, the excitation parameters must meet the stability boundary requirements, and the excitation voltage must be as small as possible. When the whole-angle control of the HRG has double-frequency, $\omega_{\min}^{\;}=\omega_{0}^{\;}$, the excitation parameters meet the following conditions:

(13) $$\begin{array}{c} \Delta_{\min}=0\\ s_{\min}=\frac{2\omega_{0}}{Q_{0}} \end{array}$$

The voltage of excitation is

(14) $$V_{1\min}=\frac{\omega_{0}^{2}m_{0}d_{0}^{2}}{4Q_{0}C_{0}V_{0}}$$

## 3. The Energy Control Scheme of the Double-Frequency Parametric Excitation

**Figure 6:**
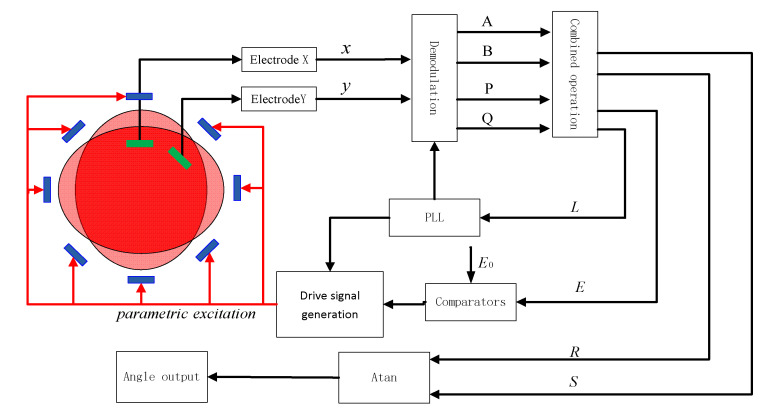
The energy control scheme of the double-frequency parametric excitation.

The energy control scheme of double-frequency parametric excitation is shown in Figure 6. The electrode of the parametric excitation is consistent with Figure 4, and the detection of signal x and y corresponds to the electrical signal, which is the output of the 0° and 45° electrodes, respectively [17].

The main components of the control system of the HRG are the loop of demodulation, the loop of the frequency and phase tracking (PLL), and the loop of the amplitude control. To obtain the decision control of the HRG, signal x (0° detection electrode) and signal y (45° detection electrode) are respectively multiplied and demodulated with the reference signal to obtain the parameters *A*, *B*, *P* and *Q*, and obtain the decision content *E*, *R*, *S* and *L* by the filter [18,19]:

(15) $$\begin{array}{l} E=A^{2}+B^{2}+P^{2}+Q^{2}\\ S=2(AB+PQ)\\ R=A^{2}-B^{2}+P^{2}-Q^{2}\\ L=2(AP+BQ) \end{array}$$

where *E* is the total vibration energy of the resonator in the current system, and the error of the energy control will be obtained by the difference between *E* and the target value of energy control $E_{0}$. The adjustment of the vibration energy is realized by the PI(Proportional-Integral) control. *L* is used to track the frequency and phase of the resonator. *S* and *R* can express the azimuth of the vibration and the azimuth is:

(16) $$\theta =\frac{1}{4}atan\left(R/S\right)$$

## 4. Simulation of the Energy Compensation of Double-Frequency Parametric Excitation by the Discrete Electrode

To verify the feasibility of the energy compensation of double-frequency parametric excitation by discrete electrode, the resonator with different excitation parameters was simulated to determine the evolution of the resonator and the control method with different excitation parameters. In addition, although reference [13] has proven that the standing wave can move freely with parametric excitation, it has not yet been proven that the energy of the standing wave is constant in free precession. The paper proposed the determination of total energy stability change of the resonator in free precession by simulation.

### 4.1. The Simulation Parameter

A high-precision HRG has strict requirements regarding structure and electrical characteristics. The typical structure and electrical parameters of the HRG in the simulation is shown in Table 1.

Inserting the parameter values into Equations (13) and (14), the optimal driving frequency and voltage are:

(17) $$\begin{array}{c} \omega_{\min}=4500*2\pi \;\mathrm{rad}/s\\ V_{1\min}=8.37\;V \end{array}$$

### 4.2. The Evolutionary Simulation of the Resonator with Different Excitation Parameters

**Figure 7:**
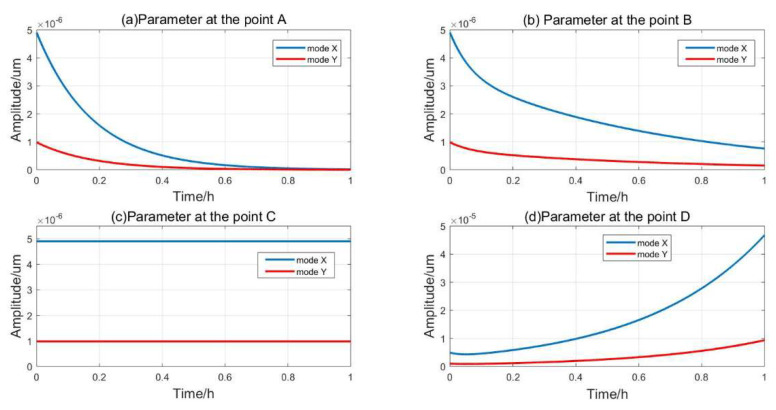
The amplitude evolution of resonator with different excitation parameter, (**a**) $\omega =4500*\frac{2\pi \mathrm{rad}}{s},V_{1}=0\;V$, the resonator vibration attenuates freely; (**b**)$\;\omega =4500*\frac{2\pi \mathrm{rad}}{s},V_{1}=5\;V$, the resonator vibration attenuates but slower; (**c**) $\omega =4500*\frac{2\pi \mathrm{rad}}{s},V_{1}=8.37\;V$, resonator vibration keeps constant; (**d**) $\omega =4500*\frac{2\pi \mathrm{rad}}{s},V_{1}=11\;V$, the resonator vibration grows exponentially.

From Figure 5, four points A, B, C, and D are taken to illustrate the vibration of the resonator. The amplitude evolution with different excitation parameters is simulated by Equations (7) and (9), as shown in Figure 7.

If $\omega =4500*\frac{2\pi \mathrm{rad}}{s},V_{1}=0\;V$—the excitation condition shown by point A in Figure 5—the resonator is not excited and vibration of the resonator will be free to attenuated to 0 due the influence of damping, as shown in Figure 7a. If $\;\omega =4500*\frac{2\pi \mathrm{rad}}{s},V_{1}=5\;V$, then $V_{1}<V_{1\min}$—the excitation parameter is point B of the attenuation region I in Figure 5—the energy supplement cannot offset the energy dissipation caused by damping, and the vibration amplitude of the resonator is still attenuated to 0, as shown in Figure 7b. If $\omega =4500*\frac{2\pi \mathrm{rad}}{s},V_{1}=8.37\;V$, then $V_{1}=V_{1\min}$, and the excitation condition is the stability boundary point C in Figure 5; the energy supplement and dissipation are equal, and the x-mode and y-mode both maintain a stable amplitude, as shown in Figure 7c. If $\omega =4500*\frac{2\pi \mathrm{rad}}{s},V_{1}=11\;V$, then $V_{1}>V_{1\min}$, the excitation condition is point D in the unstable region II in the Figure 5. The energy supplement is higher than the energy dissipation and the amplitude of the resonator will increase exponentially, as shown in Figure 7d.

**Figure 8:**
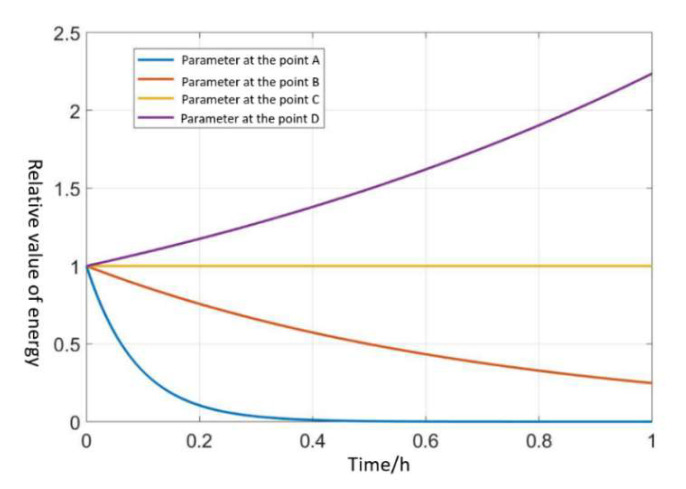
The ratio of total energy to the initial total energy with different excitation parameters.

The ratio of total energy to the initial total energy with different excitation parameters is shown in Figure 8:

(1) In the state of S = 0 (i.e., V1 is 0 voltage), the resonator will be freely damping; even if the resonator is vibrating, it will eventually decay to 0.
(2) In region I, the vibration of the resonator will gradually decline to 0; that is, the supplementary energy of the resonator is not enough to maintain the vibration of the resonator.
(3) In region II, the vibration of the resonator remains constant and the energy of the resonator is equal to the energy consumed.
(4) In region III, the vibration of the resonator will increase gradually; the supplementary energy is higher than that necessary to maintain the vibration of the resonator.

According to the simulation results, the PI control is used to realize the energy compensation of the resonator, so that the vibration of the resonator works on the amplitude required by the HRG. After the oscillation of the resonator, the PI control will cut off the starting circuit and connect the control circuit of the parametric excitation. If the initial amplitude is less than the target value, the excitation parameter is in area III, and the amplitude will gradually increase to the target value. If the initial amplitude is larger than the target value, the excitation parameter is in region I, and the amplitude will gradually decay to the target. Therefore, irrespective of the value of the initial amplitude, the final energy of the resonator will be stable to the target value by the whole-angle control, which is designed in this paper.

### 4.3. Total Energy Stability Simulation of the Resonator with the Parametric Excitation

The total energy E of the resonator is proportional to the vibration amplitude of x and y; $E=A^{2}+P^{2}+B^{2}+Q^{2}$, where E is used to represent the change of the total energy.

**Figure 9:**
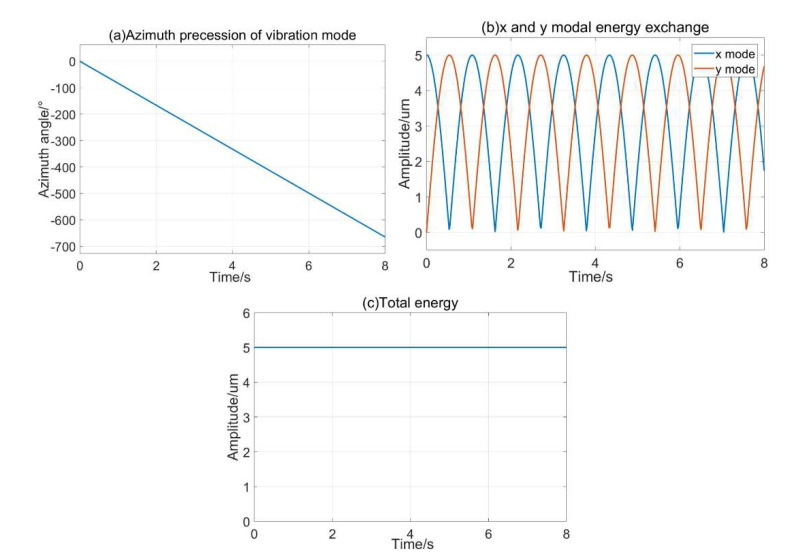
The vibration state of the resonator at 300°/s angular velocity (**a**) azimuth precession; (**b**) modal energy exchange process; (**c**) total energy during rotating.

The vibration state of the resonator with parametric excitation investigated when the angular velocity Ω≠0. If the input angular velocity is 300°/s, the azimuthal angle of the resonator moves the free precession with time. The energy exchanged between the x-mode and y-mode, and the total energy of the resonator remains unchanged during the installation process. The simulation results are shown in Figure 9. Thus, the energy compensation of the resonator can be realized when the standing wave moves the free precession, and the dynamic range of the HRG is improved compared to the force-to-rebalance.

## 5. The Experiments and Analysis

### 5.1. The Experimental Device

**Figure 10:**
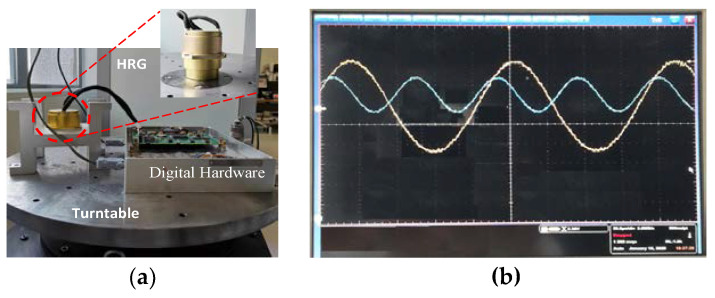
The hardware system and the parametric excitation of the HRG: (**a**) HRG and hardware system; (**b**) parametric excitation signal and detection signal x.

To verify the control scheme in Section 2, the hardware system was designed and implemented, as shown in Figure 10. The HRG used in this experiment is a typical three-suite structure, as shown in Figure 10a, which is composed of the resonator, the forcing house and the detection base. The spherical surfaces of the detection base and the forcing house are both covered with a metal film layer, and after laser etching, excitation electrodes and detection electrodes are formed, respectively. The hemispherical resonator is the core of the HRG and made of fused silica, of which the diameter is 30 mm. After chemical corrosion, vacuum annealing, balancing, metallization coating and vacuum packaging, the hemispherical resonator in HRG has a very high value of Q, and the value of Q is more than 7 million. The hardware system of the HRG with the parametric excitation has consisted of the interface of signal detection hardware, the interface of field excitation, the system of the digital hardware system based on Field Programmable Gate Array (FPGA), and the peripheral circuit. The interfaces of the signal detection hardware and field excitation are two parts of an analog circuit system, which is used to realize the signal interaction between the system and the HRG. The system of digital hardware based on FPGA is a digital circuit system, which is used to realize the control of the gyro workflow, the implementation of digital signal processing and control algorithms, and the functions of peripheral communication. The peripheral circuit includes the power supply, the communication chip, the device of protection and isolation, etc. The HRG and digital hardware board were placed on a high-precision turntable. Parametric excitation and the vibration are observed by the oscilloscope as Figure 10b.

### 5.2. The Experimental Results and Analysis

The energy compensation of the resonator by the parametric excitation was verified in the laboratory. In the actual circuit design, the vibration signal of the resonator is converted into a voltage signal by capacitor–voltage (C–V) conversion. Therefore, instead of the amplitude, the voltage corresponding to the amplitude can be chosen to represent the vibration energy of the resonator. First, the detection voltage corresponding to the amplitude of the resonator was excited to 2.3 V by the position excitation. Then, the position excitation was cut off, and the parametric excitation signal was connected for amplitude control. Finally, the target value of parametric excitation was set to E_0_ = 2.500 V and the excitation signal frequency was set equal to twice the vibration signal.

**Figure 11:**
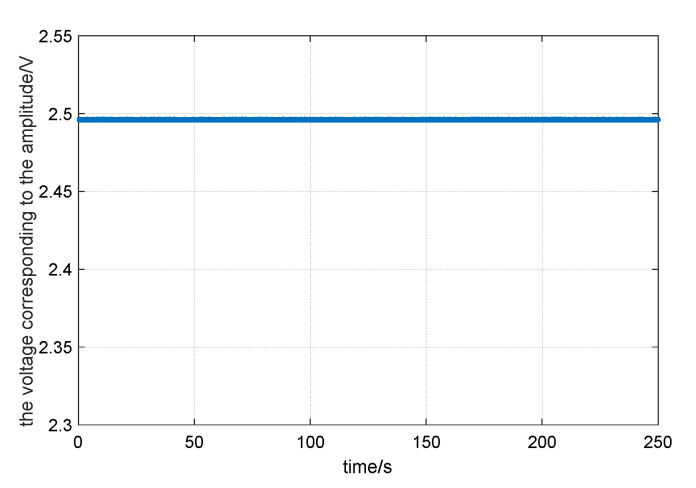
The energy compensation result of the resonator in a static state, the resonator vibration energy was constant under the double-frequency parametric excitation.

After the total energy of the vibration has stabilized, the data of detection voltage were collected; the time of collection was 250 s. The change of detection voltage with time is shown in Figure 11. At this time, the average of the detection voltage corresponding to the amplitude was E = 2.496 V; this was a horizontal straight line, and did not decay with time.

It is shown that the dampening effect was eliminated by the parametric excitation, and the energy compensation of the resonator was realized when it was in a static state.

**Figure 12:**
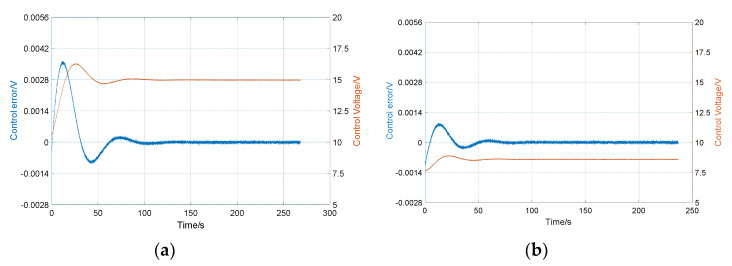
Energy compensation results: (**a**) the single-frequency parametric excitation, in which the control voltage is 15.1 V; (**b**) the double-frequency parametric excitation, in which the control voltage is 8.5 V.

The control effect of the single-frequency parametric excitation and the double-frequency parametric excitation in the experiment are shown in Figure 12. Figure 12a shows the result of single-frequency parametric excitation. When the energy is stable, the control voltage is 15.1 V. Figure 12b shows the result of double-frequency parametric excitation. When the energy is stable, the control voltage is only 8.5 V. Clearly, the control voltage of energy compensation under double-frequency parametric excitation is lower than single frequency parametric excitation. Therefore, when parametric excitation is used to maintain the energy vibration of the resonator, the double-frequency method has higher energy compensation efficiency.

It can be seen from Figure 12 that there is a high frequency noise in the error of amplitude control. The variance level of this noise is about 0.0004 V, which is 1.6 × 10^−4^ of the total amplitude. The ratio of the x-mode and y-mode amplitude is angular resolution, so this error will directly cause the angle measurement noise. The source of high-frequency noise is more complicated, and it may come from environmental vibration, temperature changes, and control system errors. In order to improve the measurement accuracy of the gyro, the noise must be further analyzed and suppressed.

**Figure 13:**
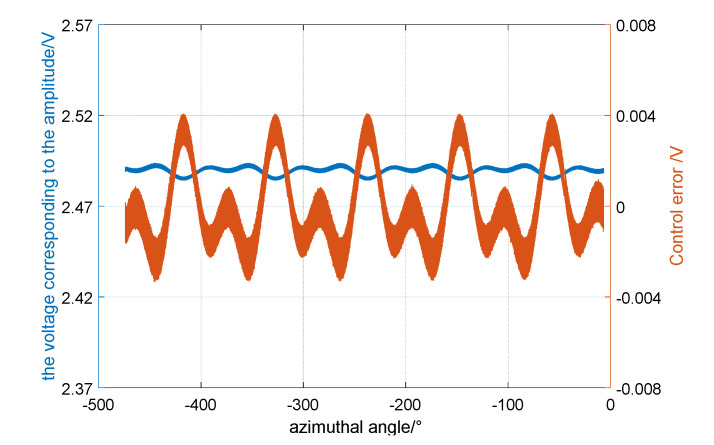
The energy compensation results of the resonator in a rotating state. The control error change periodically changed with the azimuth angle of the standing wave.

Then, the energy compensation effect of parametric excitation was observed when the angular velocity was input. The experimental device was fixed on the high-precision turntable, as shown in Figure 10, and the turntable speed was set to 300°/s. When the energy control was stabilized, the energy and the error of the energy change with different azimuths of the standing wave during rotations are shown in Figure 13. This proves that parametric excitation can still achieve the energy compensation of the HRG when there are external rotations because the average value of the energy in the 360° range remains stable.

However, it can also be seen from Figure 13 that the control error of energy periodically changes with the azimuth angle of the standing wave, indicating that the effect of energy control is different in different directions. This may be caused by the existence of non-uniform factors such as frequency-mismatch [20,21,22] and damping-mismatch [23] in the HRG. In the future, it will be necessary to add the loop of the control and optimize the control algorithm to suppress the energy fluctuation when the standing wave rotates [24,25,26].

**Figure 14:**
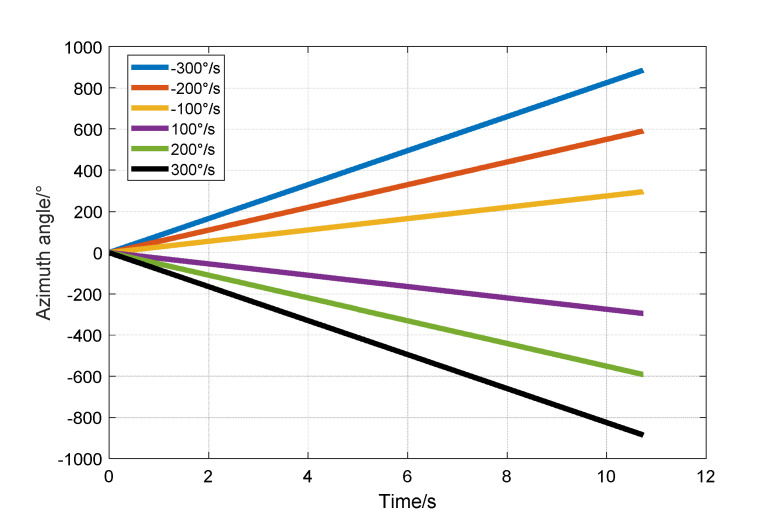
The standing waves at different angular velocities by the double-frequency parametric excitation. The standing wave azimuth are all linear precessions.

Finally, it was verified that parametric excitation of the HRG in the whole-angle control has the function of angular velocity integral measurement. Using the experimental device shown in Figure 10, the speed of the turntable was set to 300, 200, 100, −100, −200 and −300, as shown in Figure 14. The standing waves are linear precessions at different angular velocities.

After linear fitting of the angular velocity of precession, the velocity of the standing wave precession and the scale factor of the precession at different external angular speed inputs are shown in Table 2. The precession factor at different angular velocities is consistent with the theoretical precession factor −0.277, based on the hemispherical shell mode in reference [13]. This shows that the HRG has a whole-angle control under parametric excitation and has the function of angular velocity integral output.

**Figure 15:**
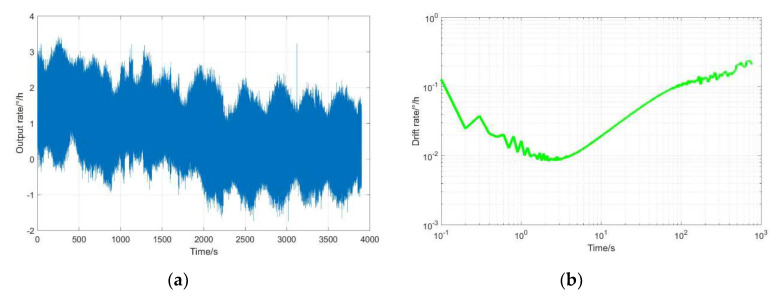
The measurement results after mathematical compensation with parameter excitation and its evaluation: (**a**) angular velocity output; (**b**) Allan variance.

In order to measure the bias instability (BI) and angle random walk (ARW) of the HRG, the output axis of the HRG points to the sky and measures 1 h of static data. For the HRG, the drift rate has a sinusoidal relationship with the azimuth of the standing wave. Based on this characteristic, the static output data are compensated mathematically, and the result is shown in Figure 15; the BI is 0.008°/h and the ARW is 0.00027°/$\sqrt{h}$.

The experimental results show that the energy compensation of the resonator was successfully achieved by the double-frequency parametric excitation, and the standing-wave exhibits free precession. Thus, it can be used to achieve energy compensation in the whole-angle control of the HRG by double-frequency parametric excitation with discrete electrodes.

## 6. Conclusions

In this paper, the dynamic model and stability boundary of the HRG based on the double-frequency parametric excitation with discrete electrode are described in detail.

The optimal excitation parameters of the HRG are given and simulated to provide the basis for energy compensation of the parametric excitation. In addition, it was shown that the total energy of the resonator remains stable during the free precession of the standing wave. The scheme of the control is designed, and energy compensation of the HRG with double-frequency parametric excitation of the discrete electrode was realized. The measurement results show that the HRG is in the whole-angle control with double-frequency parametric excitation of the discrete electrode, thereby significantly improving the dynamic range of the HRG compared with the force-to-rebalance significantly.

Through the theoretical model and experimental results described in this paper, a parametric excitation method with double frequency is provided for the hemispherical resonant gyroscope with only discrete electrodes. It will also help to achieve whole-angle control in the two-suite gyro and provide a basis for error analysis.

## Figures and Tables

**Table 1:** Typical structure and electrical parameters of a hemispherical resonator gyro.

$C_{0}\left(pF\right)$	$\omega_{0}^{\;}\left(rad/s\right)$	$d_{0}\left(um\right)$	$V_{0}\left(V\right)$
**3**	4500 × 2π	100	100
$m_{0}(g)$	$Q_{0}$	**Modal x initial amplitude (um)**	**Modal y initial amplitude (um)**
**2.2**	7,200,000	5	1

**Table 2:** The velocity of the standing wave precession and the scale factor of the precession at different external angular velocity inputs.

External Input Angular Velocity	Standing Wave Precession Angular Velocity	Precession Scale Factor
−300°/s	82.76°/s	−0.2758
−200°/s	55.10°/s	−0.2755
−100°/s	27.57°/s	−0.2757
100°/s	−27.56°/s	−0.2756
200°/s	−55.28°/s	−0.2764
300°/s	−82.84°/s	−0.2761

## Appendix A

From reference [14], after considering the excitation electrode, the dynamic equation of the resonator can be obtained as:

(A1) $$\begin{array}{c} m\ddot{q_{1}}+kq_{1}-\frac{C_{0}}{2d_{0}^{3}}\overset{N}{\underset{i=1}{\sum }}V_{i}^{2}{\left(d_{0}+q_{1}cos2\theta_{i}+q_{2}sin2\theta_{i}\right)}^{2}cos2\theta_{i}=0\\ m\ddot{q_{2}}+kq_{2}-\frac{C_{0}}{2d_{0}^{3}}\overset{N}{\underset{i=1}{\sum }}V_{i}^{2}{\left(d_{0}+q_{1}cos2\theta_{i}+q_{2}sin2\theta_{i}\right)}^{2}sin2\theta_{i}=0 \end{array}$$

Replacing *q*1 and *q*2, respectively, by the symbols x and y used in this paper yields:

(A2) $$\begin{array}{c} m\ddot{x}+kx-\frac{C_{0}}{2d_{0}^{3}}\overset{N}{\underset{i=1}{\sum }}V_{i}^{2}{\left(d_{0}+xcos2\theta_{i}+ysin2\theta_{i}\right)}^{2}cos2\theta_{i}=0\\ m\ddot{y}+ky-\frac{C_{0}}{2d_{0}^{3}}\overset{N}{\underset{i=1}{\sum }}V_{i}^{2}{\left(d_{0}+xcos2\theta_{i}+ysin2\theta_{i}\right)}^{2}sin2\theta_{i}=0 \end{array}\;$$

Thus:

(A3) $$\begin{array}{c} F_{x}=\frac{C_{0}}{2d_{0}^{3}}\overset{N}{\underset{i=1}{\sum }}V_{i}^{2}{\left(d_{0}+xcos2\theta_{i}+ysin2\theta_{i}\right)}^{2}cos2\theta_{i}\\ F_{y}=\frac{C_{0}}{2d_{0}^{3}}\overset{N}{\underset{i=1}{\sum }}V_{i}^{2}{\left(d_{0}+xcos2\theta_{i}+ysin2\theta_{i}\right)}^{2}sin2\theta_{i} \end{array}\;$$

In the HRG, since the amplitudes *x* and *y* are much smaller than the gap $d_{0}$ (the ratio of the amplitude to the gap is usually 1/10–1/20), the above formula is expanded and only the first-order quantity is retained, thus:

(A4) $$F_{x}=\frac{C_{0}}{2d_{0}^{3}}\overset{N}{\underset{i=1}{\sum }}V_{i}^{2}\left(d_{0}^{2}+2d_{0}xcos2\theta_{i}+2d_{0}ysin2\theta_{i}\right)cos2\theta_{i}\;F_{y}=\frac{C_{0}}{2d_{0}^{3}}\overset{N}{\underset{i=1}{\sum }}V_{i}^{2}\left(d_{0}^{2}+2d_{0}xcos2\theta_{i}+2d_{0}ysin2\theta_{i}\right)cos2\theta_{i}$$

## Appendix B

To illustrate why $d_{0}^{2}$ in Equation (3) and $V_{0}^{2},{\left(V_{1}\cos2\omega t\right)}^{2}$ in Equation (4) do not affect the energy compensation of double-frequency parametric excitation, take the x-mode as an example. The equivalent effect of the electrode driving on the x-mode is:

(A5) $$\begin{array}{c} F_{x}=\frac{C_{0}}{2d_{0}^{3}}\overset{N}{\underset{i=1}{\sum }}V_{i}^{2}(d_{0}^{2}+2d_{0}xcos2\theta_{i}+2d_{0}ysin2\theta_{i})cos2\theta_{i}\\ F_{y}=\frac{C_{0}}{2d_{0}^{3}}\overset{N}{\underset{i=1}{\sum }}V_{i}^{2}(d_{0}^{2}+2d_{0}xcos2\theta_{i}+2d_{0}ysin2\theta_{i})cos2\theta_{i} \end{array}$$

and:

(A6) $$\begin{array}{c} x=\mathrm{Acos}\omega t+\mathrm{Psin}\omega t\\ y=\mathrm{Bcos}\omega t+\mathrm{Qsin}\omega t \end{array}$$

Therefore, the equivalent power of the equivalent driving force is:

(A7) $$P_{x}=F_{x}\dot{x}\;$$

and the power of each vibration cycle by the driving force is:

(A8) $$W_{x}=\int_{0}^{2\pi /\omega }P_{x}dt\;$$

After integrating the above formula, then $d_{0}^{2}$ in Equation (3) and $V_{0}^{2},{\left(V_{1}\cos2\omega t\right)}^{2}$ in Equation (4) will be equal to zero. The same result can be derived for the y mode. Thus, $d_{0}^{2}$ in Equation (3) and $V_{0}^{2},{\left(V_{1}\cos2\omega t\right)}^{2}$ in Equation (4) do not affect the energy compensation of double-frequency parametric excitation.

## Appendix C

The dynamic equation of the hemispherical resonator gyro is:

(A9) $$\{\begin{array}{c} \ddot{x}-4K\Omega \dot{y}+\frac{\omega_{0}}{Q_{0}}\dot{x}+\omega_{0}^{2}x=\delta cos2\omega tx\\ \ddot{y}+4K\Omega \dot{x}+\frac{\omega_{0}}{Q_{0}}\dot{y}+\omega_{0}^{2}y=\delta cos2\omega ty \end{array}$$

Let:

(A10) $$\begin{array}{c} x=\mathrm{Acos}\omega t+\mathrm{Psin}\omega t\\ y=\mathrm{Bcos}\omega t+\mathrm{Qsin}\omega t \end{array}$$

Since A, B, P, and Q are slow variables, $\dot{A},\dot{B},\dot{P},\dot{Q},\ddot{A},\ddot{B},\ddot{P},\ddot{Q}$ can all be ignored; only their product term with ω needs to be retained. Then:

(A11) $$\begin{array}{c} \dot{x}=-A\omega \sin\omega t+P\omega \cos\omega t\\ \dot{y}=-B\omega \sin\omega t+Q\omega \cos\omega t \end{array}$$

(A12) $$\begin{array}{c} \ddot{x}=-{A\omega }^{2}\cos\omega t-{P\omega }^{2}\sin\omega t-2\dot{A}\omega \sin\omega t+2\dot{P}\omega \cos\omega t\\ \ddot{y}=-{B\omega }^{2}\cos\omega t-\mathrm{Qsin}\omega t-2\dot{B}\omega \sin\omega t+2\dot{Q}\omega \cos\omega t \end{array}$$

Take the first equation in Equation (6) as an example. After substitution, the equation becomes:

(A13) $$\begin{array}{c} -{A\omega }^{2}\cos\omega t-{P\omega }^{2}\sin\omega t-2\dot{A}\omega \sin\omega t+2\dot{P}\omega \cos\omega t-4K\Omega \left(-B\omega \sin\omega t+Q\omega \cos\omega t\right)\\ +\frac{\omega_{0}}{Q_{0}}\left(-A\omega \sin\omega t+P\omega \cos\omega t\right)+\omega_{0}^{2}\left(\mathrm{Acos}\omega t+\mathrm{Psin}\omega t\right)\\ =\delta \cos2\omega t\left(\mathrm{Acos}\omega t+\mathrm{Psin}\omega t\right)\\ =\frac{1}{2}\delta \left(A\left(\cos3\omega t+\cos\omega t\right)+P\left(\sin3\omega t-\sin\omega t\right)\right)\; \end{array}$$

Ignoring the high-frequency terms, using the coefficient equalization method, we obtain:

(A14) $$\begin{array}{c} -{A\omega }^{2}+2\dot{P}\omega -4K\Omega Q\omega +\frac{\omega_{0}}{Q_{0}}P\omega +\omega_{0}^{2}A=\frac{1}{2}\delta A\\ -{P\omega }^{2}-2\dot{A}\omega +4K\Omega B\omega -\frac{\omega_{0}}{Q_{0}}A\omega +\omega_{0}^{2}P=-\frac{1}{2}\delta P \end{array}$$

That is:

(A15) $$\begin{array}{c} \dot{P}=-\frac{1}{2}\frac{\omega_{0}}{Q_{0}}P-\frac{1}{2}\left(\frac{\omega_{0}^{2}-\omega^{2}}{\omega }-\frac{1}{2}\frac{\delta }{\omega }\right)A+2K\Omega Q\\ \dot{A}=\frac{1}{2}\left(\frac{\omega_{0}^{2}-\omega^{2}}{\omega }+\frac{1}{2}\frac{\delta }{\omega }\right)P-\frac{1}{2}\frac{\omega_{0}}{Q_{0}}A+2K\Omega B \end{array}$$

Similarly, we can derive:

(A16) $$\begin{array}{c} \dot{Q}=-\frac{1}{2}\frac{\omega_{0}}{Q_{0}}Q-\frac{1}{2}\left(\frac{\omega_{0}^{2}-\omega^{2}}{\omega }+\frac{1}{2}\frac{\delta }{\omega }\right)B-2K\Omega P\\ \dot{B}=\frac{1}{2}\left(\frac{\omega_{0}^{2}-\omega^{2}}{\omega }-\frac{1}{2}\frac{\delta }{\omega }\right)Q-\frac{1}{2}\frac{\omega_{0}}{Q_{0}}B-2K\Omega A \end{array}$$

Thus:

(A17) $$\{\begin{array}{c} \dot{P}=-\frac{1}{2}\left(\Delta -\frac{1}{2}s\right)A-\frac{1}{2}\frac{\omega_{0}}{Q_{0}}P+2K\Omega Q\\ \dot{A}=-\frac{1}{2}\frac{\omega_{0}}{Q_{0}}A+\frac{1}{2}\left(\Delta +\frac{1}{2}s\right)P+2K\Omega B\\ \dot{Q}=-\frac{1}{2}\left(\Delta -\frac{1}{2}s\right)B-\frac{1}{2}\frac{\omega_{0}}{Q_{0}}Q-2K\Omega P\\ \dot{B}=-\frac{1}{2}\frac{\omega_{0}}{Q_{0}}B+\frac{1}{2}\left(\Delta +\frac{1}{2}s\right)Q-2K\Omega A \end{array}$$

where $\Delta =\frac{\omega_{0}^{2}-\omega^{2}}{\omega },s=\frac{\delta }{\omega }$.

## References

[1] Sciences A., Rozelle D., Grumman N., Rozelle D. The Hemispherical Resonator Gyro: From Wineglass to the Planets. Proceedings of the 19th AAS/AIAA Space Flight Mechanics Meeting.

[2] Anthony M. The Operation and Mechanization of the Hemispherical Resonator Gyroscope. Proceedings of the IEEE/ION PLANS.

[3] Jeanroy A., Grosset G., Goudon J.C., Delhaye F. HRG by Sagem from laboratory to mass production. Proceedings of the IEEE International Symposium on Inertial Sensors and Systems.

[4] Jeanroy A., Bouvet A., Remillieux G. (2014). HRG and marine applications. Gyroscopy Navig..

[5] Ragot V., Remillieux G. (2011). A New Control Mode Greatly Improving Performance of Axisymmetrical Vibrating Gyroscopes. Gyroscopy Navig..

[6] Delhaye F. HRG by SAFRAN: The game-changing technology. Proceedings of the 2018 IEEE International Symposium on Inertial Sensors and Systems (INERTIAL).

[7] Xia Y., Qi Y.N., Cai X. (2018). Method to reduce angular increment error of hemispherical resonators unit by time management. Fligth Control. Detect..

[8] Wang X., Wu W., Fang Z., Luo B., Li Y., Jiang Q. (2012). Temperature Drift Compensation for Hemispherical Resonator Gyro Based on Natural Frequency. Sensors.

[9] Zhao H.B., Ren S.Q., Teng H.J. (2013). Establishment of steady state model of amplitude-control for hemispherical resonator gyro under force-rebalance mod. J. Chin. Inert. Technol..

[10] Gregory J.A., Cho J., Najafi K. Novel mismatch compensation methods for rate-integrating gyroscopes. Proceedings of the 2012 IEEE/ION Position, Location and Navigation Symposium.

[11] Loper E., Lynch D.D. (1979). Sonic Vibrating Bell Gyro. U.S. Patent.

[12] Loper E., Lynch D.D. (1990). Vibratory Rotation Sensor. U.S. Patent.

[13] Matveyev V., Lunin B., Basarab M. (2009). Solid-State Wave Gyro.

[14] Zhuravlev V. (2004). Drift of An Imperfect Hemispherical resonator gyro. Mech. Solids.

[15] Wei Z., Yi G., Huo Y., Qi Z., Xu Z. (2019). The Synthesis Model of Flat-Electrode Hemispherical Resonator Gyro. Sensors.

[16] Nayfeh A.H., Mook A.D. (2008). Nonlinear Oscillations.

[17] Zhang L.X., Zhao W.L., Li S.L., Chen Y.S., Wang W. (2019). Signal Processing and Control Method of Whole Angle Mode Hemispherical Resonator Gyros. Navig. Position. Timing.

[18] Lynch D.D. Coriolis Vibratory Gyros. Symposium Gyro Technology. Proceedings of the Symposium Gyro Technology.

[19] Lynch D.D. Vibratory gyro analysis by the method of averaging. Proceedings of the 2nd International Conference on Gyroscopic Technology and Navigation.

[20] Lynch D.D. MRIG frequency mismatch and quadrature control. Proceedings of the 2014 International Symposium on Inertial Sensors and Systems (ISISS).

[21] Wang Y., Pan Y., Qu T., Jia Y., Yang K., Luo H. (2018). Decreasing Frequency Splits of Hemispherical Resonators by Chemical Etching. Sensors.

[22] Li S.L., Yang H., Xia Y. (2020). Measurement Method of Hemispherical Resonator Frequency Splitting and Normal-mode Axis Azimuth Based on Amplitude Frequency Response Characteristics. Fligth Control. Detect..

[23] Pan Y., Qu T., Wang D., Wu S., Liu J., Tan Z., Yang K., Luo H. (2017). Observation and analysis of the quality factor variation behavior in a monolithic fused silica cylindrical resonator. Sens. Actuators A Phys..

[24] Zhbanov Y.K. Self-tuning control loop for suppression of quadrature in a hemispherical resonator gyro. Proceedings of the Saint Petersburg International Conference on Integrated Navigation Systems.

[25] Zhbanov Y.K. (2008). Amplitude control contour in a hemispherical resonator gyro with automatic compensation for difference in Q-factors. Mech. Solids.

[26] Shatalov M., Coetzee C. (2011). Dynamics of Rotating and Vibrating Thin Hemispherical Shell with Mass and Damping Imperfections and Parametrically Driven by Discrete Electrodes. Gyroscopy Navig..

